# An Inexpensive AI-Powered IoT Sensor for Continuous Farm-to-Factory Milk Quality Monitoring

**DOI:** 10.3390/s25144439

**Published:** 2025-07-16

**Authors:** Kaneez Fizza, Abhik Banerjee, Dimitrios Georgakopoulos, Prem Prakash Jayaraman, Ali Yavari, Anas Dawod

**Affiliations:** Department of Computing Technology, Swinburne University of Technology, Melbourne, VIC 3122, Australia; abanerjee@swin.edu.au (A.B.); pjayaraman@swin.edu.au (P.P.J.); ayavari@swin.edu.au (A.Y.); adawod@swin.edu.au (A.D.)

**Keywords:** Internet of Things, in-tank milk monitoring, IoT sensor

## Abstract

The amount of protein and fat in raw milk determines its quality, value in the marketplace, and related payment to suppliers. Technicians use expensive specialized laboratory equipment to measure milk quality in specialized laboratories. The continuous quality monitoring of the milk supply in the supplier’s tanks enables the production of higher quality products, better milk supply chain optimization, and reduced milk waste. This paper presents an inexpensive AI-powered IoT sensor that continuously measures the protein and fat in the raw milk in the tanks of dairy farms, pickup trucks, and intermediate storage depots across any milk supply chain. The proposed sensor consists of an in-tank IoT device and related software components that run on any IoT platform. The in-tank IoT device quality incorporates a low-cost spectrometer and a microcontroller that can send milk supply measurements to any IoT platform via NB-IoT. The in-tank IoT device of the milk quality sensor is housed in a food-safe polypropylene container that allows its deployment in any milk tank. The IoT software component of the milk quality sensors uses a specialized machine learning (ML) algorithm to translate the spectrometry measurements into milk fat and protein measurements. The paper presents the design of an in-tank IoT sensor and the corresponding IoT software translation of the spectrometry measurements to protein and fat measurements. Moreover, it includes an experimental milk quality sensor evaluation that shows that sensor accuracy is ±0.14% for fat and ±0.07% for protein.

## 1. Introduction

The dairy supply chain involves multiple stakeholders, including dairy farms, logistics providers, and milk processing plants, who often operate independently with limited visibility into the live status of milk quality at other stages. A key concern is the condition of raw milk at the farm, which significantly influences decision-making throughout the supply chain. Because of this, raw milk is typically assessed on various characteristics, such as organoleptic, compositional, physical, chemical, and hygienic, which are the key categories. The early detection of milk quality issues is critical to prevent spoilage during storage and pickup. However, current practices rely mainly on cooling regulations and manual inspections, such as smell tests before collecting, due to the lack of live monitoring in farm vats and transport vehicles [1]. Milk processors are particularly interested in milk fat and protein content, as these parameters affect both farmer payments and production planning for products like high-protein dairy goods [2]. Currently, milk quality is assessed through the laboratory testing of samples collected during pickup, which not only brings in additional costs but also introduces delays and limits the ability to make timely decisions. The absence of live milk quality data restricts processors from optimizing production planning based on incoming milk characteristics [3,4].

Integrated dairy supply chains can help improve the efficiency of milk processing and lead to greater sustainability [1]. As milk fat and protein composition play an important role in decision-making at dairy plants [2], having live access to information about incoming milk quality from farms can help dairy plants adapt and optimize production. This is particularly because the fat and protein content of raw milk received from milk farms can impact the nutritional value of dairy products as well as production efficiency, with the type of impact varying depending on the type of product. For instance, the fat and protein composition in milk has been shown to impact both the quality of yogurt [3,4], as well as the incubation time. Similarly, the protein-to-fat ratio of raw milk is also shown to impact the quality of cheese production [5]. Thus, prior knowledge of the milk quality at farms can enable milk processing plants to better plan production. Further, this information can also help improve production efficiency. For instance, due to the correlation between the composition of raw milk and product quality, milk processing plants typically standardize milk before production. Taking the example of cheese production, [6,7] identified the impact of milk protein content on the final quality of the produced cheese. Thus, an awareness of milk composition at dairy farms can also help improve the standardization process, which is undertaken in all milk processing plants [8].

However, despite the benefits of live access to the quality of milk stored at dairy farms for production planning and optimization, existing systems for milk quality measurement on farms are designed to work only on milking lines to measure milk from individual cows. This is not useful for milk processors, although the quality of milk stored in vats is the aggregate of all milk on a farm. Further, the milk quality is also impacted by storage conditions, such as the cooling systems. On the other hand, quality control methods used in testing laboratories and milk processing plants rely on desktop analysers that are bulky and too costly for individual farms [5]. Existing research solutions and commercial devices for sensing milk quality primarily focus on laboratory-based or inline processing environments rather than on-farm storage or transportation. These solutions often rely on benchtop spectrometers, biosensors, or chemical analysis kits that require sample collection and external testing, leading to delays in quality assessment. While some inline sensors are integrated into dairy processing facilities, they are not designed for continuous live monitoring in storage tanks at farms or in mobile transportation systems.

The ability to monitor milk quality inside storage tanks on farms is crucial because milk undergoes biochemical changes due to temperature fluctuations, bacterial growth, and enzymatic activity. Further, milk fat and protein content, as well as other quality parameters, are influenced by a variety of factors, including environmental conditions, implying that the quality of milk can vary quickly on the same farm. Thus, in the absence of the live monitoring of milk quality on farms, milk processing plants lack any information that allows them to adapt production planning to the quality of incoming milk. Similarly, milk transportation lacks information that can allow them to optimize pick-up routes. Existing solutions fail to address these challenges, as they are not equipped for deployment in dynamic and potentially harsh environments, such as milk tanks and milk trucks.

In this paper, we present an AI-based milk quality sensor to enable the live monitoring of milk in tanks in milk supply chains. Specifically, we developed a milk quality sensor suitable for deployment in milk tanks on dairy farms, pickup trucks, and depots across any milk supply chain. The AI-enabled smart milk quality sensor incorporates a spectrometer and LEDs connected to an Arduino microcontroller [6]. While the use of spectroscopy has been explored in the literature for measuring milk fat and protein content, existing approaches use high-end expensive spectrometers and focus on identifying and utilizing specific spectral channels for fat or protein measurement. In the proposed sensor, we use all available spectral channels to achieve high accuracy even when using a low-cost spectrometer with limited bandwidth and low resolution. This is achieved by combining the low-cost sensor that can be economically deployed in thousands of suppliers with an ML-based translator. In addition, the design of the proposed IoT-based milk quality sensor allows it to be easily incorporated into virtually any existing IoT platform. The main contributions of the paper are as follows.

- An inexpensive milk quality monitoring system at milk farms that continuously measures the fat and protein content of raw milk in the tanks of the milk suppliers, the pickup trucks and depots across any milk supply chain. It combines commercially available IoT hardware with ML.
- An in-tank IoT device that combines an inexpensive spectrometer, LED lights, an IoT Arduino microcontroller, and an MB-IoT network card. The device houses these electronics in a food-safe, semi-transparent polypropylene enclosure, which is specifically fabricated for milk tanks.
- An ML algorithm that helps translate spectroscopy milk measurements to fat and protein measurements.
- An experimental evaluation of the proposal using hundreds of samples of raw milk from different dairy farms shows that the proposed sensor provides a measurement accuracy of ±0.14% for fat and ±0.07% for protein.

The rest of the paper is organized as follows. Section 2 discusses the related literature, while Section 3 introduces the materials and methods, where we present the developed milk quality sensor, its architecture, in-tank device component, and its software module. Section 4 presents the performance evaluation results of our developed sensor, and Section 5 concludes the paper, presenting some future research directions for this work.

## 2. Related Work

Ensuring milk quality across the dairy supply chain has long been a priority for both producers and processors. Currently, milk quality assessment in the dairy supply chain depends heavily on periodic sampling and laboratory testing and relies on regulatory compliance through cooling protocols for ensuring the suitability of consumption. Tests on raw milk from farms, which analyze various characteristics including fat and protein content, are often conducted post-pickup and used for pricing and production decisions. While certain physical, chemical, and hygienic characteristics determine the suitability of milk for consumption, compositional characteristics such as fat and protein determine the nutritional value and processing suitability of the milk and are also used for farmer payment [9]. In the remainder of the paper, unless otherwise stated, we will use the term milk quality to refer to compositional characteristics, focusing on fat and protein content.

Existing milk testing methods are limited by delays in obtaining results, a lack of live insight, and inefficiencies in production planning due to a lack of compositional awareness at the farm and transport stages. In [10], the authors reviewed different rapid methods, sensors, and commercial systems for chemical and hygienic characteristics such as milk spoilage and microorganism detection. The major focus lies on chemical sensors and biosensors for the detection of the established indicators associated with bacterial growth and milk spoilage, like changes in pH value, conductivity/impedance, adenosine triphosphate (ATP) level, the concentration of consumed O_2_ and produced CO_2_, and volatile and non-volatile compounds. In addition, wireless sensors and colour indicators for intelligent packaging are discussed. Some other similar works have explored the use of portable or low-cost spectrometers for milk quality estimation [9]. However, they are time-consuming and not suitable for live decision-making. Existing on-farm solutions, such as those integrated into automated milking systems (e.g., DeLaval and Lely), focus on per-cow analysis during milking sessions and fail to provide aggregated quality metrics for milk stored in bulk vats, which is of primary interest to milk processors [11].

Chakraborty and Biswas [12,13] focused on milk adulteration and why it is crucial to detect such alterations. In their study, they identified the minimum detectable limit of adulterants in milk and proposed a low-cost sensor and a detection system to detect the fat content in milk. The proposed sensor is evaluated on milk with a fat content of 3%, 6%, and 12% and shown to have 0.5% accuracy. This makes it unsuitable for measuring the quality of raw milk at farms, which have much finer variation in fat and protein. The authors in [6] highlighted the importance of measuring the quality of raw and processed milk. They also discussed various existing approaches for measuring the milk quality via measuring dry matter, protein, fat, and lactose. However, these approaches are aimed at detecting milk spoilage but do not look to measure live milk quality, specifically the ability to continuously monitor the fat and protein composition of raw milk stored in milk tanks.

Advanced dairy processing plants deploy inline sensors, such as Fourier Transform Infrared (FTIR) spectrometers and biosensors, to analyze milk composition in real-time. While these devices offer high accuracy, they are expensive, large, and unsuitable for deployment in remote, rural, or mobile contexts like milk vats on farms or in pickup trucks [14]. However, these approaches do not demonstrate suitability for deployment in dairy farms as they are either limited to supermarket milk or are limited to standalone quality evaluations [15]. Further, they do not capture quality degradation due to improper storage or transport conditions between the farm and the plant.

To address the limitations of lab-based methods, researchers have explored portable sensing technologies. For instance, Zhang et al. [16] developed a portable FTIR system for on-site milk quality assessment, and Wang et al. [17] proposed a multi-spectral sensor for estimating protein and fat content. Although these approaches demonstrate promise, they typically use only a subset of spectral channels and require precise calibration or ideal testing environments, limiting their robustness for real-world applications.

The emergence of the Internet of Things (IoT) has introduced new possibilities for the live monitoring of agricultural and food systems. IoT-enabled milk monitoring systems have been developed to track temperature, milk levels, and cow health, but the integration of compositional sensors (e.g., for fat and protein estimation) into these platforms remains limited. Milk Moovement [18], for example, offers the cloud-based tracking of logistics and quality metrics, but still depends on delayed lab testing. Recent industry trends highlight a growing interest in using sensor networks and wireless technologies to enable proactive decision-making across dairy supply chains [19].

Deploying sensors for milk quality in on-farm and transport environments requires overcoming several challenges, including temperature fluctuations, vibrations, and the risk of contamination. Most commercial and academic solutions are not designed for these harsh and dynamic conditions and are too expensive (example: HiYi LactoScope 300 FT-IR is listed for USD 100,000 at https://www.alibaba.com/product-detail/HiYi-LactoScope-300-FT-IR-Dairy-1600819813520.html, accessed on 9 July 2025). Additionally, current portable devices typically lack the necessary sensitivity and specificity to monitor milk composition continuously in bulk volumes. For instance, in [20], the authors conducted a lab-based evaluation of portable near-infrared spectroscopy (NIR) devices for distinguishing different types of supermarket milk with fat content varying by at least 0.5% from each other. However, raw milk stored at dairy farms is characterized by a much more fine-grained variance of compositional characteristics, typically around 0.01%, which needs to be monitored continuously.

Despite the recognized importance of fat and protein composition for product quality and production efficiency [21,22] existing solutions largely fail to provide reliable, live compositional data from storage tanks on farms or in transit. This gap limits the ability of processors to adapt and optimize production schedules based on incoming milk quality.

Our work addresses these gaps by proposing an AI-based smart milk quality sensor that integrates a full-spectrum spectrometer with machine learning algorithms for accurate fat and protein estimation. Unlike the existing works that often focus on single-purpose sensing or laboratory testing, our sensor is designed for robust, live use in farm and transport settings. Furthermore, our system enables continuous quality tracking, enhancing the potential for adaptive and sustainable dairy supply chain management. To the best of our knowledge, this is the first work on developing a low-cost IoT-based milk quality sensor for live milk monitoring in dairy supply chains.

## 3. Milk Quality Sensor System Design

This section presents the design of the proposed milk quality sensor, including the materials and methods used for this. More specifically, Section 3.1 describes the sensor architecture. The in-tank IoT device component of the sensor system is presented in Section 3.2, and the IoT software component of the milk quality sensor system that includes the ML translator is presented in Section 3.3.

### 3.1. Milk Quality Sensor System Architecture

**Figure 1:**
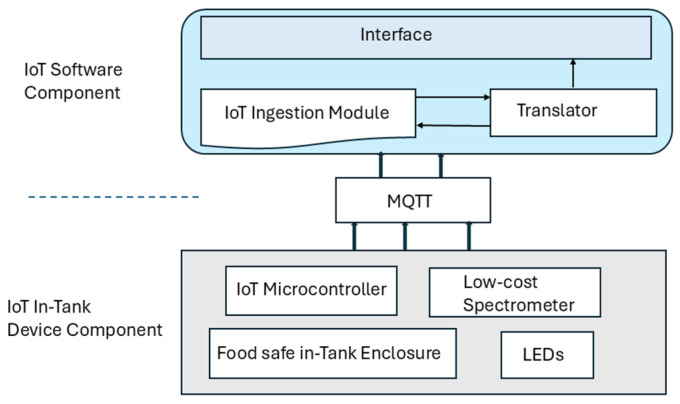
Architecture of developed milk quality sensor.

In this section, we present the architecture of our milk quality sensor system (MQSS), which can be used via any available IoT platform. The milk quality sensor system consists of two main components that are depicted in Figure 1: the in-tank IoT device and IoT software component. The dairy industry requires (1) a milk quality sensor that is inexpensive, as it has to be deployed in hundreds of thousands of dairy farms, pickup trucks, and deports, and (2) ready for use via any IoT platform they have available, as this enables low-cost integration, and the use of the milk quality measurements in other industry applications, such as milk supply forecasting, pick-up scheduling, etc. To keep the in-tank IoT device inexpensive, the in-tank IoT component of the sensor is designed to utilize commercially available hardware components that are integrated to provide continuous milk spectroscopy measurements to any IoT platform. The detailed design of the in-tank IoT component is presented in Section 3.2. The IoT software component of the milk quality sensors is an IoT application that includes an ML algorithm (i.e., a generic term for referring to any machine learning algorithm used for estimating fat and protein content) for translating milk spectroscopy data to protein and fat measurements.

Please note the following:

- The proposed milk quality sensor system and its components can be easily deployed and used via any available IoT platform in the market. The in-tank IoT device and the IoT software components communicate via the MQTT protocol over narrowband (NB-IoT). The sensors in-tank and software components are described in more detail in Section 3.1 and Section 3.2, respectively.
- The proposed sensor can easily adapt to ensure other milk quality parameters, such as somatic cell count (SCC), lactose, and Solids Not Fat (SNF), by using a different converter. This will require retraining the ML algorithm by pairing the relevant ground truth data with the same milk sensor observations to generate the corresponding training dataset, which can then be used to develop an appropriate ML translator to replace the ones used for milk fat and protein here.

### 3.2. In-Tank IoT Device Component

**Figure 2:**
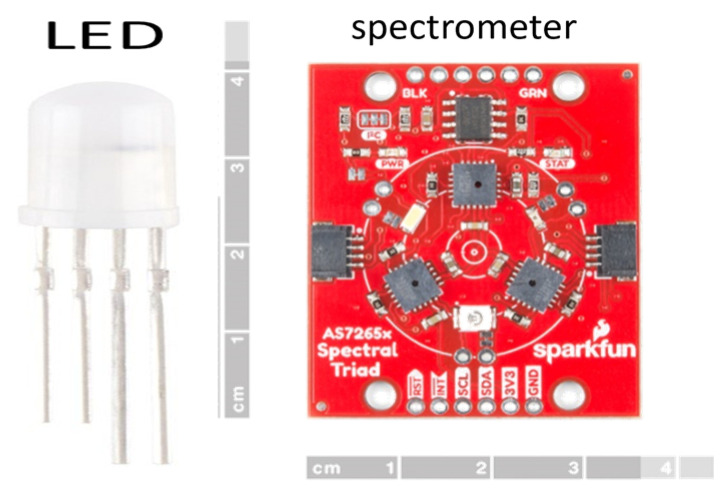
LED and spectrometer used in the developed milk quality sensor.

**Low-cost spectrometer**: The AS7265x spectrometer costs less than USD 100 and offers a suitable spectroscopy solution for measuring milk quality, as this sensor can detect multiple bands simultaneously [23]. With 18 narrowband spectral channels covering the 410–940 nm range, this spectrometer enables a detailed analysis of spectroscopy measurements associated with milk composition, such as fat, protein, and water content. Its compact form factor and digital interface (I^2^C/UART) make it suitable for integration into portable or embedded systems, facilitating real-time, on-farm, and in-tank spectroscopy measurements. This spectrometer’s affordability and versatility present an attractive alternative to traditional high-cost laboratory spectrometers, enabling a wider adoption of spectral sensing technologies in milk quality control applications. Along with the spectrometer, we have used a Light-Emitting Diode (LED), which emits light in the visible spectrum. The LED and the spectrometer are set up at a short distance (~1 cm) that allows the passage of milk, and the measured spectra on all 18 channels are recorded as the MSU measurements. The LED and spectrometer are shown in Figure 2.

**IoT microcontroller:** For collecting the milk spectrometer measurements and sending this data to any IoT platform, we selected and utilized an MKR NB 1500 device equipped with an NB-IoT network card. The Arduino firmware is responsible for initializing the sensor and periodically capturing spectral readings (the programming for obtaining spectral readings is based on publicly available code from SparkFun, the manufacturer of the spectrometer. https://github.com/sparkfun/SparkFun_AS7265x_Arduino_Library, accessed on 9 July 2025), preprocessing raw data (e.g., noise filtering and normalization), formatting the data into CSV payloads, and sending data via NB-IoT connection to the IoT platform using MQTT.

**Food-safe in-tank enclosure/housing:** To house the above electronic component in the milk tanks of the farms, pickup trucks, and supply chain depots, we designed and custom-manufactured food-safe enclosures made from polypropylene. A primary motivation is for the device to be used for the continuous in situ monitoring of milk quality in tanks at dairy farms, and it is designed to be cleaned as part of the regular clean-in-place (CIP) cycles of milk tanks. Thus, the choice of material needs to be able to withstand the chemicals used for CIP, as well as the temperature range of milk tanks, for which the maximum temperature does not exceed 80 °C during clean-in-place (CIP) cycles, while staying below 5 °C for stored milk. The polypropylene we used is food-contact approved as per U.S. Food and Drug Administration (FDA) regulations [24], tolerant to chemicals used for tank washing [25], and with an operating temperature range from less than 0 °C to greater than 100 °C, thus making it suitable for use in milk tanks. In addition, the food-safe polypropylene material and lack of exposed joins prevent milk supply contamination. The presence of exposed joins can lead to a buildup of milk residue, leading to contamination. A buildup of milk residue over time also leads to contamination. To prevent this, the enclosure is washed automatically by the tank’s automatic cleaning system after every milk pick-up and, more thoroughly, manually during periodic tank maintenance.

**Figure 3:**
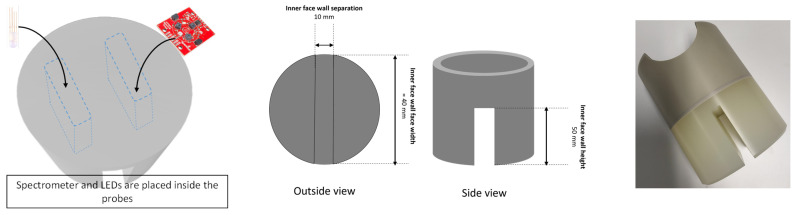
Design of the polypropylene-based sensor enclosure, including dimensions, and after fabrication.

The enclosure for the milk quality sensor consists of two probes, one of which houses the spectrometer and the other houses the LED, as shown in Figure 2. As also shown in Figure 3, the dimensions of each of the faces of the probes are 40 mm × 50 mm, and the separation between the probes is 10 mm. To achieve the correct operation of the milk quality sensor, the probes (containing the spectrometer and LED) need to be immersed completely in milk. Given the dimensions of the probes, this implies a minimum of 20 mL of milk. The actual minimum volume of milk, however, would depend on the dimensions of the milk tank, which ensures at least 20 mL of milk between the probes See Figure 3.

### 3.3. IoT Software Component

Apart from the hardware component that houses the spectrometer and IoT microcontroller, we discuss, in Section 3.2, the milk quality sensor which includes a software component that can run as an IoT application in any available IoT platform. The software component of the milk quality sensor includes the following modules:

**IoT ingestion module**: This ingests the spectrometry measurements that are continuously collected from the milk supply via the in-tank IoT device spectrometer and sends it to the translator module for converting spectrometry data to protein and fat measurements. The IoT microcontroller in the tank and the IoT platform communicate via NB-IoT using the MQTT (Message Queueing Telemetry Transport)—a common protocol supported by virtually all existing IoT platforms. MQTT automatically stores the spectrometry data it receives from the IoT microcontroller of each milk quality sensor in a different queue. The MQTT queues are automatically created and managed by the IoT platform based on the number of in-tank IoT devices that have been registered in the IoT platform and the spectrometry measurements that are generated by each in-tank IoT device. The IoT ingestion modules access the queue of each in-tank IoT device, dequeue its spectroscopy data, and forward it to the translator module.

**Translator:** This module takes live milk spectrometry data as input, and with the help of a trained ML algorithm, it translates the input spectrometry data into milk fat and protein measurements. The ML algorithm is trained with data from over six hundred milk quality report data from a laboratory that specializes in testing the milk supply. The following steps were taken to train the underlying ML algorithm that is incorporated in the translator module.

1. **Creation of dataset for ML algorithm training**: To create the ML algorithm training dataset, we collected fresh farm milk samples directly from dairy farms. The actual fat and protein contents of these samples were determined through independent laboratory testing, which served as ground truth data. Simultaneously, spectral measurements were captured using an in-tank IoT device in a university lab setting and correlated with the ground truth data to create the training dataset for the ML algorithm.
2. **Training and evaluating the ML algorithm**: We experimented with multiple ML algorithms, including decision trees, random forests, and linear regression. Separate ML algorithms were trained for estimating fat and protein values, with the spectral measurements corresponding to the 18 channels as the input features. These algorithms were evaluated using rigorous testing and cross-validation techniques to identify the best-performing algorithm. The results showed that while decision tree and random forests are promising, linear regression emerged as the most accurate and reliable method for predicting fat and protein content in raw milk due to its ability to handle the variations in fat and protein content in farm milk.
3. **Deploying the ML algorithm**: After training and evaluation, we incorporated the optimized linear regression algorithm in our IoT software component of the proposed milk quality sensor. The spectral measurements are read every 1 s. Each spectral measurement is provided as input to the “Translator” component, allowing for milk quality values to be updated every second.

This ML algorithm training process permits the following:

- The continuous ingestion of spectral measurements into the trained machine learning algorithm, which produces instant predictions of fat and protein content, enabling the rapid and reliable quality assessment of milk.
- The periodic retraining of the ML algorithm using a larger number and variety of samples that will allow further improvement in the accuracy and resilience of the milk quality sensor.

## 4. Milk Quality Sensor System Evaluation

To evaluate the milk quality sensor system, we integrated its in-tank IoT device and IoT software components in a commercially available IoT platform, i.e., Software AG’s Cumulocity [26].

To experimentally validate the effectiveness of the developed milk quality sensor, we evaluated it using fresh farm milk samples. Here, we use the term ‘fresh farm milk samples’ to refer to the fact that the tests using the milk samples were conducted in a manner to ensure the milk quality did not change from the time of sample collection, by following industry-approved processes. For this, (a) milk samples were obtained directly from dairy farms before further processing, (b) milk samples were stored in specially cooled containers which maintained temperatures below 5 °C, and (b) tests using the milk quality sensor were conducted within 8 h of the samples being picked up. To establish the ground truth for training the ML algorithm, milk samples were also tested at a commercial lab that uses Fourier Transform Infrared (FTIR) spectroscopy to measure fat and protein content in milk, which is the standard method of determining milk composition in the dairy industry [27]. Further, the commercial lab chosen for FTIR was one that also undertakes milk testing for milk from the same dairy farms as part of existing supply chain processes, thus ensuring consistency. The experimentation steps for testing milk samples and training and the evaluation of the ML algorithm are detailed as follows:

1. Collected more than 600 pairs of raw milk samples from more than 80 dairy farms across Victoria, Australia. Each pair of samples was marked with a unique identifier.
2. Sent the first of the first sample from each farm to our laboratory and the second milk sample to BVAQ—a commercial lab that is used by the Australian dairy industry to test their milk supply.
3. Used the in-tank IoT device to measure the fat and protein content of the milk samples we send to our laboratory. Over 600 samples were tested this way.
4. BVAQ provided their milk testing reports for the samples they measured the protein and fat content using their specialized laboratory process, machines, and technicians. The over 600 milk testing reports we received from BVAQ provided the ground truth.
5. Correlated the protein and fat measurement we obtained from the in-tank IoT device in (3) and the ground truth from the BVAQ milk testing reports in (4) using the unique identifier from (1).
6. Trained the ML algorithm using the correlated dataset in (5).
7. Assessed the accuracy of the translator of the milk quality sensor using the remaining data from (3) that were not used in training the ML algorithm.

From the ground truth data, based on the milk testing reports, the protein content in the milk samples was found to vary between 2.89% and 4.43%, with a median value of 3.53%. The fat content had a minimum value of 1.84% and a maximum of 6.34% with a median of 4.58%.

To evaluate the performance of the ML algorithm, we measured the classification accuracy error as the difference between the milk quality (fat and protein) classification/estimation of the milk quality sensor and the milk quality ground truth from the BVAQ milk analysis reports. We evaluated multiple machine learning algorithms for estimating fat and protein content, using a train–test split of 80:20%. The performance of four different machine learning algorithms, namely linear regression, decision tree (DT) regression, random forest regression, and K-Nearest Neighbours (KNNs), is compared in the Table 1, which shows the mean estimation error over 10 iterations. While classification algorithms may be suitable for distinguishing supermarket milk, which typically has fixed fat and protein content, they are less appropriate for farm milk, where fat and protein content can vary as mentioned above. Classifying such variations would require a large number of samples to represent each specific fat and protein level (e.g., 2.0%, 2.1%, etc.). To handle this complexity, we used linear regression algorithms in our evaluation.

As noted earlier, we found that linear regression had the best performance on the 600 pairs of samples we used in this evaluation as it achieved the smallest classification/estimation error for both fat and protein, with average classification/estimation errors as 0.14% for fat and 0.07% for protein composition. The coefficients of the linear regression algorithm can be found in Table A1 in Appendix A. On the other hand, DT regression had the lowest accuracy for fat with 0.21%, while KNN regression had the lowest accuracy for protein composition.

**Figure 4:**
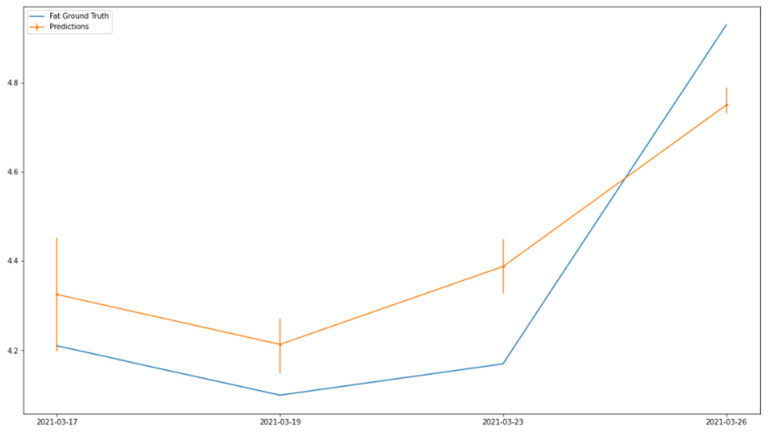
Fat trend comparison over time.

**Figure 5:**
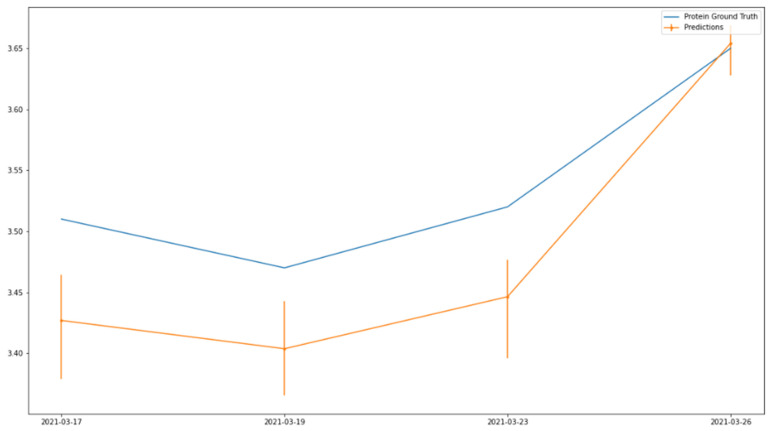
Protein trend comparison over time.

To further illustrate the usefulness of the milk quality sensor, we look at how the milk quality sensor can be used to monitor milk quality trends on individual farms. This is because trends and forecasts of milk production at individual farms have already been explored for the optimization of pick-up routes, which can be enhanced by including milk quality as part of such route planning [28,29]. Figure 4 and Figure 5 show samples of milk fat and protein classifications/estimation together with the ground truth for the same farm over multiple days, demonstrating not only the same trend but also closely matching milk quality values.

The estimation error can be reduced further by training the algorithms using additional milk samples.

## 5. Conclusions and Future Research

In this paper, we proposed, developed, and evaluated an inexpensive AI-based IoT sensor system. The developed system is unique in a way that it can be deployed inside a milk tank. This feature allows the use of the developed sensor in tanks at farms, pick-up trucks, and depots across any milk supply chain, eliminating the cost and time of collecting, shipping, and analyzing milk samples in remote industrial milk analysis labs. Moreover, the hardware used to develop this sensor is low-cost and hence does not limit its deployment in each milk tank. Further, it addresses the challenge of measuring live milk quality, i.e., the developed system containing the software component helps to measure the milk quality in real-time, without bringing the milk to laboratories and using heavy machines to measure the quality of milk offline. The developed sensor system shows that sensor accuracy is ±0.14% for fat and ±0.07% for protein.

In this paper, our focus is on developing a minimal cost milk quality sensor system which can be deployed inside a milk tank. Despite the contribution, it is possible to extend the evaluation of the developed sensor system in different scenarios, such as improving the process of manufacturing more complex milk products, such as cheese-making.

## Figures and Tables

**Table 1:** Comparison of machine learning algorithms.

Machine Learning Algorithm	Fat	Protein
Linear Regression	0.143376	0.075953
DT Regression	0.207111	0.107925
Random Forest Regression	0.179536	0.089263
KNN Regression	0.205551	0.109128

## Data Availability

No new data were created or analyzed in this study. Data sharing is not applicable to this article.

## Appendix A

In this appendix, we show the coefficients of the linear regression model of the milk fat and protein in Table A1.

**Table A1:** Coefficients of the linear regression algorithm.

Spectroscopy Channels	Fat	Protein
Ch1	0.028879	−0.02064466
Ch2	−0.02161235	0.06879262
Ch3	−0.01461645	0.03216482
Ch4	−0.00213582	0.00036747
Ch5	0.01519708	−0.05223435
Ch6	0.06608016	0.00698689
Ch7	−0.06186486	0.0209115
Ch8	−0.09539753	−0.03128069
Ch9	−0.06232195	−0.04209589
Ch10	−0.01646882	0.01167448
Ch11	0.07641843	0.01654799
Ch12	0.02896871	0.00783537
Ch13	−0.0186936	0.00219042
Ch14	−0.09573338	−0.0127194
Ch15	−0.11991515	−0.02106304
Ch16	−0.03782334	0.02359651
Ch17	−0.00463765	0.00677463
Ch18	−0.045751	−0.01391183
Intercept	6.22671706	2.7979688
